# Treatment of refractory pemphigus vulgaris with efgartigimod

**DOI:** 10.1016/j.jdcr.2025.04.012

**Published:** 2025-04-25

**Authors:** Matthew Helm, Gracyn Allan, Brigid Deck, Galen Foulke

**Affiliations:** a Department of Dermatology, Penn State Health Milton S. Hershey Medical Center, Hershey, Pennsylvania; b Penn State College of Medicine, Hershey, Pennsylvania; c Department of Public Health Sciences, Penn State College of Medicine, Hershey, Pennsylvania

**Keywords:** efgartigimod, pemphigus, refractory pemphigus vulgaris

## Introduction

Pemphigus vulgaris (PV) is an autoimmune disorder characterized by IgG autoantibodies to desmoglein 3 and/or desmoglein 1, essential components of the desmosomes, which bind keratinocytes. IgG-mediated dysfunction of desmoglein results in weakened intracellular adhesion, providing an explanation for the deep epidermal and oral mucosal blistering seen in PV.^1^ Efgartigimod is an engineered Fc fragment antagonist of neonatal Fc receptor, which is vital in IgG recycling. Inhibition of this pathway serves to significantly decrease circulating levels of IgG.^2^ In the United States, efgartigimod has been approved since 2021 for use in generalized myasthenia gravis, an IgG-mediated autoimmune neuromuscular disease, and has been investigated since then for use in other IgG-driven autoimmune diseases. Among these are immune thrombocytopenia, autoimmune myositis, and pemphigus disorders.^3^

Standard treatment for rapid control of moderate to severe PV is usually achieved with use of systemic steroids with or without rituximab (an anti-CD20 therapy) or disease modifying antirheumatic drugs.^4^ Efgartigimod has been shown in a recent phase 2 clinical trial to improve the condition of patients with PV.^5^ We report a case of refractory PV in which treatment with efgartigimod resulted in sustained remission.

## Case report

**Fig 1:**
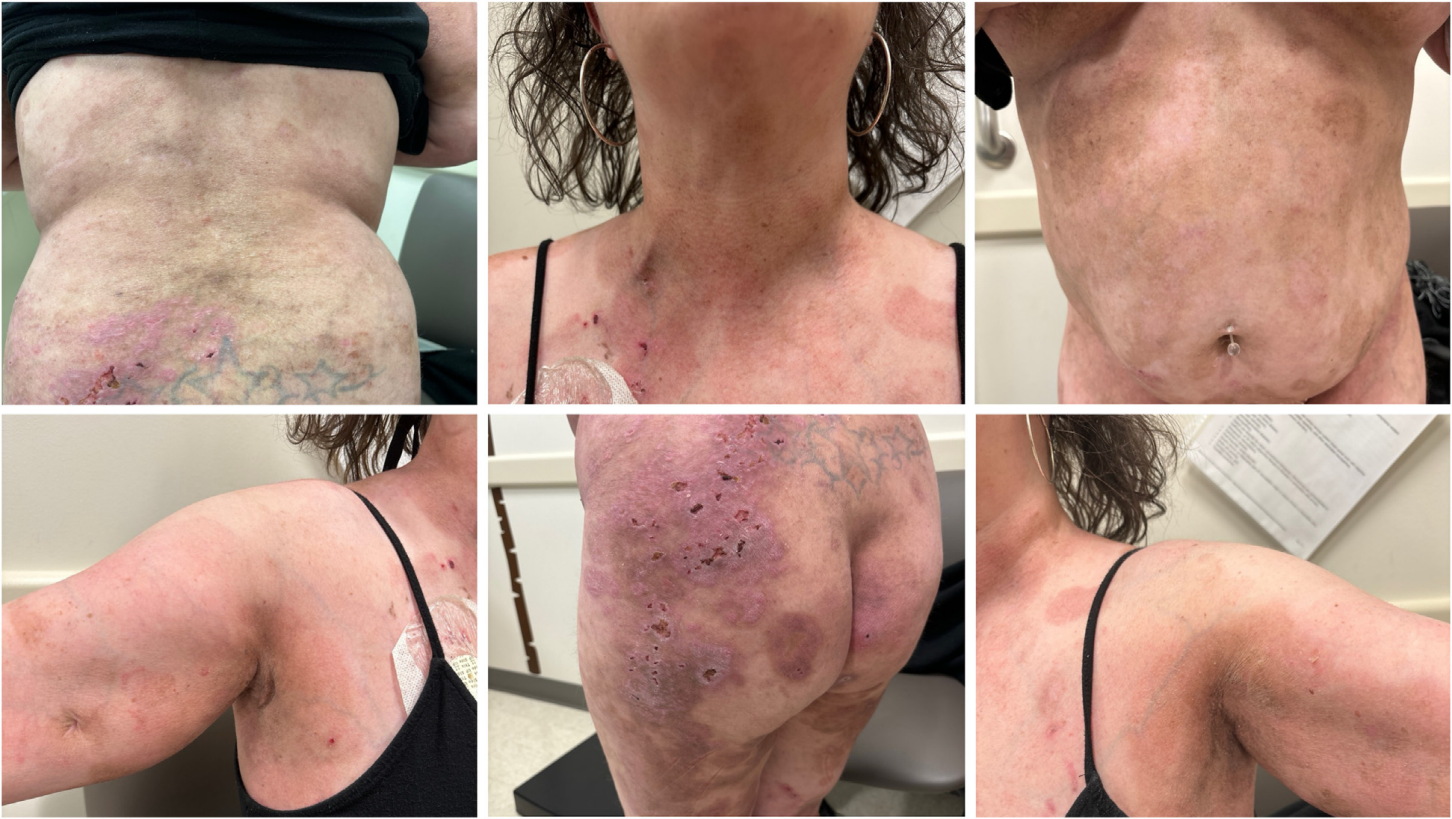
Pemphigus vulgaris after plasmapheresis.

A 38-year-old woman presented with poorly controlled PV impacting approximately 60% body surface area (Fig 1). The patient was diagnosed with PV in 2019, although likely experienced symptoms prior. She had antibodies for both desmoglein 1 and desmoglein 3. On physical examination, widespread erosions were noted around the eyes, mouth, armpits, buttocks, lower portion of the legs, and in the intertriginous areas, in addition to severe stomatitis. The patient was experiencing dysphagia, oral ulcers, memory loss, feeling cold, and an inability to tolerate putting on clothes due to excessive pain. She denied shortness of breath, fevers, or chills. She had malignancy screening 2 years prior, which was unremarkable. The presenting flare was preceded by a few missed doses of cyclophosphamide. The patient was ultimately admitted to the intensive care unit to treat her severe PV, secondary infections of the skin, and chronic pain.

The patient had been refractory to many therapies, including cyclophosphamide (75 mg daily), steroid tapers (20-100 mg daily), mycophenolate mofetil (1500 mg twice daily), azathioprine (100 mg daily), methotrexate (20 mg weekly), intravenous immunoglobulin (2 g/kg every 4 weeks), and dapsone (75 mg daily). She experienced anaphylaxis to rituximab 3 times including a desensitization protocol. During her intensive care unit admission she responded well to 5 rounds of plasmapheresis. Ocrelizumab (anti-CD20 antibody approved for multiple sclerosis) was discussed as an option, but approval was denied by insurance.

**Fig 2:**
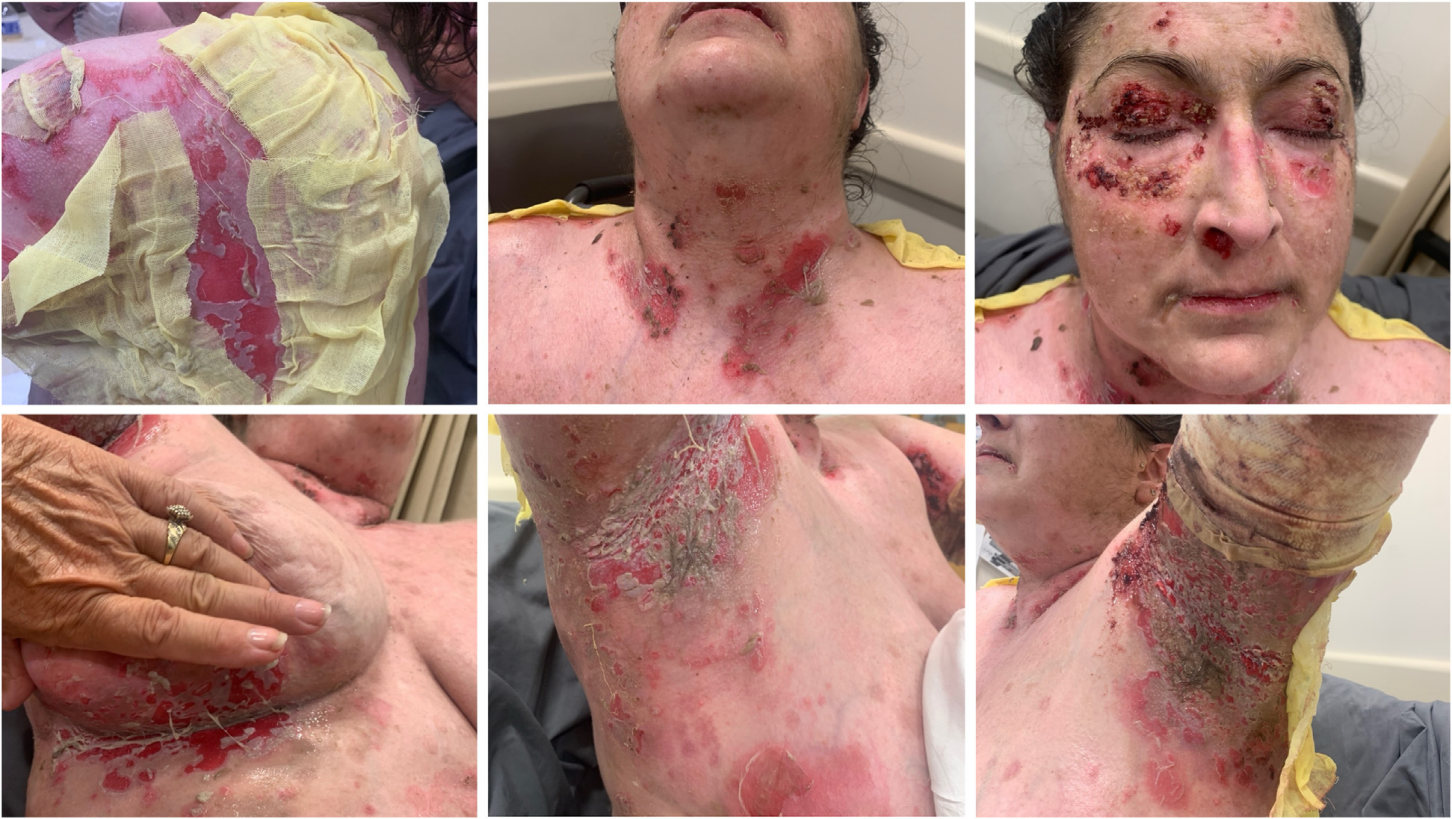
Pemphigus vulgaris initial presentation before treatment.

**Fig 3:**
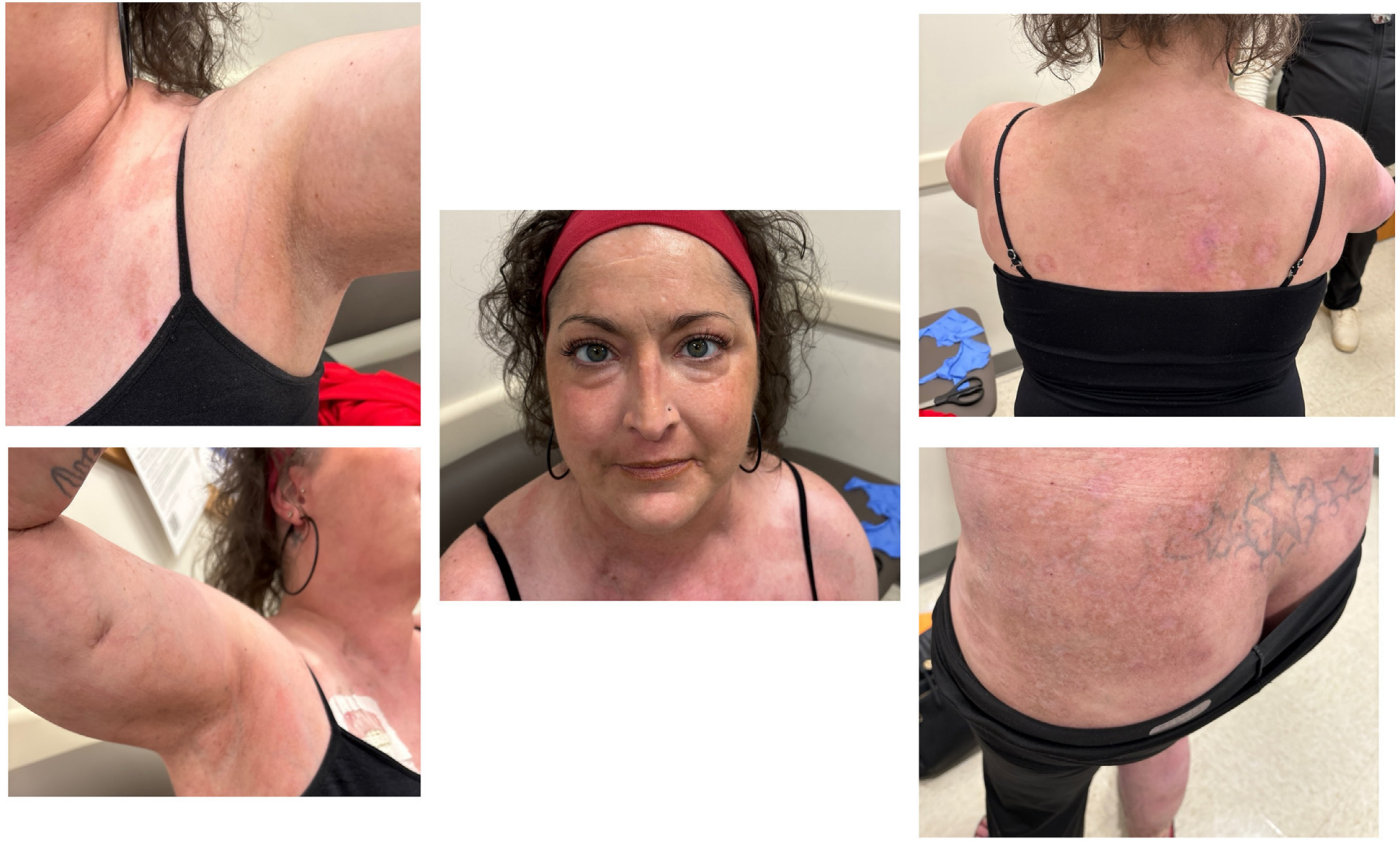
Pemphigus vulgaris 8 weeks after treatment with efgartigimod.

The decision was made to integrate efgartigimod into the treatment regimen of this patient’s severe PV. Cyclophosphamide was discontinued, and the patient was transitioned to efgartigimod. The patient received efgartigimod (10 mg/kg) infusions weekly for 4 weeks, followed by a single dose every 4 weeks based on US Food and Drug Administration-approved efgartigimod dosing for treatment of generalized myasthenia gravis.^3^ The following medications were continued: prednisone 20 mg daily, mycophenolate mofetil 1.5 g daily for oral solution, and intravenous immunoglobulin every 4 weeks. This treatment regimen was well tolerated and resulted in tremendous improvement, described by the patient as the best she had experienced in the last 4 years (Fig 2). Pemphigus Disease Area Index was 74 on initial presentation, 24 after hospitalization and plasmapheresis, and 4 after 2 doses of efgartigimod (Fig 3). The patient was able to successfully taper off prednisone, with continuation of the aforementioned medications.

Of note, the patient did experience cutaneous ulceration on bilateral calves while on this medication, which was not consistent with pemphigus and could be attributable to the efgartigimod. Ultrasound was negative for deep vein thrombosis. Tissue cultures and wound swab grew *Pseudomonas aeruginosa* and *Staphylococcus aureus*. The patient experienced cardiac complication during a procedure for these ulcers and died on September 23, 2024. Cutaneous ulceration could be a potential side effect; however, etiology was not determined, and prior reports of cutaneous ulceration due to efgartigimod have not been reported.

## Discussion

Efgartigimod’s mechanism of inhibiting the activity of neonatal Fc receptor and reducing serum IgG levels makes it a powerful tool to consider in the treatment of IgG-mediated autoimmune diseases. Although efgartigimod was initially approved for generalized myasthenia gravis, there have been a few reports discussing its efficacy in other IgG-mediated autoimmune diseases, including PV.^3^^,^^5^ A recent clinical trial investigated the effects of 10 or 25 mg/kg bodyweight intravenous efgartigimod on total levels of IgG as well as autoreactive IgG. In this open-label phase 2 adaptive trial, 34 patients with mild-to-moderate PV or pemphigus foliaceus received efgartigimod. These patients exhibited a major decrease in serum total IgG and antidesmoglein antibodies, associated with improved Pemphigus Disease Area Index scores. The treatment also works very quickly, and clinical responses have been seen in just 1 month. Overall, this study demonstrated a significant improvement in disease severity as well as demonstrating a long-term response after use of efgartigimod as monotherapy or with add on of low dose oral prednisone.^5^

To add to the array of evidence for use of efgartigimod as therapy for PV, we present in this case report an additional incidence of improvement in PV disease activity with use of efgartigimod. Our patient received efgartigimod (10 mg/kg) infusions weekly for 4 weeks, followed by a single dose every 4 weeks. We saw significant improvement in erosions of the skin and mucosa, as demonstrated in Figures 1 and 2. This outcome was cohesive with previous clinical trials, which noted decrease in serum total IgG and antidesmoglein autoantibodies along with improvement in Pemphigus Disease Area Index scores. One limitation is that titers of IgG autoantibodies to desmoglein 1 and desmoglein 3, as measured by enzyme-linked immunosorbent assay, were not recorded before and after treatment. Additional studies should include a larger sample size and capture enzyme-linked immunosorbent assay values before and after treatment to objectively assess serologic activity in response to efgartigimod.

In this case, a patient who presented with severe PV refractory to many standard therapies experienced tremendous improvement in symptoms with efgartigimod. This case contributes to the growing body of evidence demonstrating great value in further exploration of efgartigimod in the treatment of PV.

## Conflicts of interest

None disclosed.
